# Transcriptomic analysis of differentially expressed genes in the *Ras1*^CA^-overexpressed and wildtype posterior silk glands

**DOI:** 10.1186/1471-2164-15-182

**Published:** 2014-03-09

**Authors:** Li Ma, Qian Ma, Xuan Li, Leilei Cheng, Kai Li, Sheng Li

**Affiliations:** Key Laboratory of Developmental and Evolutionary Biology, Institute of Plant Physiology and Ecology, Shanghai Institutes for Biological Sciences, Chinese Academy of Sciences, Shanghai, 200032 China; School of Life Science, East China Normal University, Shanghai, 200062 China; Shanghai Institute of Cardiovascular Diseases, Zhongshan Hospital, Fudan University, Shanghai, 20032 China

## Abstract

**Background:**

Using the *piggyBac*-mediated GAL4/UAS transgenic system established in the silkworm, *Bombyx mori*, we have previously reported that overexpression of the *Ras1*^CA^ oncogene specifically in the posterior silk gland (PSG) improved cell growth, fibroin synthesis, and thus silk yield. However, the detailed molecular mechanism remains to be fully elucidated. To achieve this goal, Illumina sequencing was used in the present study to compare the transcriptomes of the *Ras1*^CA^-overexpressed and wildtype PSGs.

**Results:**

The transcriptomic sequencing results in 56 million reads following filtering steps. Most of the reads (~70%) are successfully mapped to the *Bombyx* genome. The mapped reads are situated within at least 9,133 predicted genes, covering 62.46% genes of the *Bombyx* genome. GO annotation shows that 2512 of the 2,636 differentially expressed genes (DEGs) are mostly distributed in metabolic process, cell and cell part, and binding, and KEGG annotation shows that 1,941 DEGs are mapped into 277 pathways. Importantly, *Ras1*^CA^ overexpression in the PSG upregulated many DEGs distributed in “pathways in cancer”, “insulin signaling pathway”, and “MAPK signaling pathway” as well as “purine metabolism” and “pyrimidine metabolism”. Transcriptional regulation of these DEGs was verified by quantitative real-time PCR. Moreover, injection of small-molecule chemical inhibitors of the Ras1 downstream effectors into the *Ras1*^CA^-overexpressed silkworms revealed that both Raf-MAPK and PI3K-TORC1 pathways are required for the Ras1-induced DEG expression.

**Conclusion:**

The transcriptomic analysis illustrates that, apart from phosphorylational regulation, Ras1 activates its downstream Raf-MAPK and PI3K-TORC1 pathways at the transcriptional level. Meanwhile, Ras1 increases DNA content and induces endoreplication, at least in part, by upregulating genes in “nucleotide metabolism” and “cell cycle”. This study provides further insights into the molecular mechanism of how *Ras1*^*CA*^ overexpression in the PSG improves silk yield.

**Electronic supplementary material:**

The online version of this article (doi:10.1186/1471-2164-15-182) contains supplementary material, which is available to authorized users.

## Background

As a traditional agricultural industry, sericulture is economically important to China and several other countries. The domesticated silkworm, *Bombyx mori*, is the most important insect species for sericulture. In the past, sericulture has been greatly advanced by applying the hybrid breeding technique to *Bombyx*. However, it has reached a plateau during the last decades, mostly due to the inherent threshold of this technique. To break through the bottleneck of silk production, new breeding techniques, such as the molecular breeding technique, should be developed. It has been hypothesized that fibroin production in the *Bombyx* posterior silk gland (PSG) is directly proportional to silk yield and determined by its gland size and protein synthesis [1], making it possible to improve silk yield by genetic manipulation of the PSG [2]. On the other hand, *Bombyx* has been used as a model lepidopteran insect for a long time [3, 4]. In terms of protein synthesis, its PSG is one of the most efficient organs in animals. Therefore, studying the molecular mechanism controlling fibroin synthesis in the *Bombyx* PSG is of great value for both applied and basic research.

Using the *piggyBac*-mediated GAL4/UAS transgenic system established in *Bombyx*[5–7], we specifically overexpressed a constitutively active form of *Ras1* (*Ras1*^CA^) in the PSG. In the transgenic silkworm, Fil-GAL4 > UAS-Ras1^CA^ (Fil > Ras1^CA^), *Ras1*^CA^ overexpression increases gland size and protein synthesis in the PSG, leading to silk yield improvement by 60% [2]. This study not only provides an application prospect to silk yield improvement in sericulture, but also supports the previous hypothesis that fibroin production is determined by gland size and protein synthesis in the PSG [1]. It is certain that Ras1 and its downstream Raf-MAPK and PI3K-TORC1 pathways play critical roles in regulating fibroin production [2], while the detailed molecular mechanism remains to be fully elucidated. The completed *Bombyx* genome sequence [8, 9] makes it possible to use functional genomics, such as proteomics and transcriptomics, to achieve the above goal. Using 2D-DIGE-MS/MS analysis, we previously compared the proteomic profiles of the *Ras1*^CA^-overexpressed and wild type (WT) PSGs. Further studies revealed that, via the downstream Raf-MAPK and PI3K-TORC1 pathways, *Ras1*^CA^ upregulates *bcpi*, which inhibits cathepsin activity thus preventing PSG destruction during metamorphosis [10]. Transcriptomics could be an alternative approach for analyzing how *Ras1*^*CA*^ overexpression in the PSG improves fibroin production.

In terms of transcriptomic tools, a whole-genome microarray containing 22,987 oligonucleotides of 70-mers that cover the presently known and predicted genes in the *Bombyx* genome was designed on the basis of the whole-genome sequences [11]. This microarray has been used to survey the silkworm gene expression patterns in multiple tissues, at different developmental stages, and under various conditions or treatments [11–15]. RNA-Seq (also known as Next Generation Sequencing), including Roche/454 pyrosequencing, Illumina-Solexa sequencing, and Applied Biosystems SOLiD sequencing, has led to a revolution in genomics and provided cheaper and faster delivery of sequencing information [16]. The Illumina-Solexa sequencing strategy was adopted for the sequencing of 40 *Bombyx* genomes from 29 phenotypically and geographically diverse domesticated silkworm lines and 11 wild silkworms from various mulberry fields in China. This comprehensive study constructs a genome-wide genetic variation map which shed light on the history of silkworm domestication [9]. RNA-Seq also led to the identification of new exons, novel genes, alternative splicing genes, and *trans*-splicing events in *Bombyx*[17, 18].

In this study, Illumina-Solexa sequencing revealed 2,636 differentially expressed genes (DEGs) in the *Ras1*^CA^-overexpressed and WT PSGs. Confirmed by quantitative real-time PCR (qPCR), the transcriptomic analysis shows that Ras1 increases gland size, protein synthesis, and DNA content in the PSG at the transcriptional level.

## Results

### Identification of DEGs using RNA-seq

For better understanding the molecular mechanism how *Ras1*^*CA*^ overexpression increases fibroin production in the *Bombyx* PSG, we compared the transcriptomes of the *Ras1*^CA^-overexpressed and WT PSGs at the early wandering stage. The RNA-seq raw data were deposited to NCBI SRA with the accession number SRP026709 (http://www.ncbi.nlm.nih.gov/sra/?term=SRP026709). The accession numbers for the two WT PSG RNA-seq libraries are SRX320122 and SRX320124, and those for the two *Ras1*^CA^-overexpressed PSG RNA-seq libraries are SRX320125 and SRX320126. The RNA-seq raw data of SRX320122 and SRX320124 were combined for raw data processing, so were SRX320125 and SRX320126. Using the pair-end Illumina-Solexa sequencing strategy, we obtained 69,662,027 raw reads, containing about 6.9 gigabases with an average read length of 101 nucleotides. The raw data was preprocessed, and 13,238,008 (19%) low quality reads were removed. The remaining 56,424,019 reads, with an average length of 90.71 nucleotides (Table 1), were used to map the *Bombyx* genome (release_2.0) [19] using TopHat [20].

**Table 1:** **Raw data preprocessed results**

Sample	Raw data		Valid data			Valid ratio (reads)
	Read	Base	Read	Base	Average length	
**WT**	33218225	3321822500	26436677	2385302793	90.38	79.79%
**Fil > Ras1** ^**CA**^	36443802	3644380200	29987342	2732788395	91.12	81.76%
**All**	69662027	6966202700	56424019	5118091323	90.71	81.00%

In total, 39,967,028 reads were mapped to silkworm genome database (silkgenome.fa) and silkworm gene database (silkworm_glean.gff), with approximately 2.2 and 1.8 million reads from the *Ras1*^CA^-overexpressed and WT PSGs, respectively. The completed *Bombyx* genome contains 14,623 unigenes [8, 9]. Using the Fragments Per kb Per Million Reads (FPKM) method [21], we have found that 9,133 unigenes are expressed, with 6,962 and 6,429 unigenes in the *Ras1*^CA^-overexpressed and WT PSGs, respectively. The mapping coverage is 62.46% genes of the silkworm genome, showing a high confidence of RNA-seq in this study (Table 2).

**Table 2:** **Evaluation of valid reads mapped to reference genome of each sample**

	QC data	Mapp_reads	Mapp%	Unigenes	Junc_reads	Junc%
**WT**	26436677	17583378	66.54%	6429	5694420	21.62%
**Fil > Ras1** ^**CA**^	29987342	22383650	73.35%	6962	7878212	25.33%
**All**	56424019	39967028	70.83%	9133	13572632	24.05%

**Figure 1:**
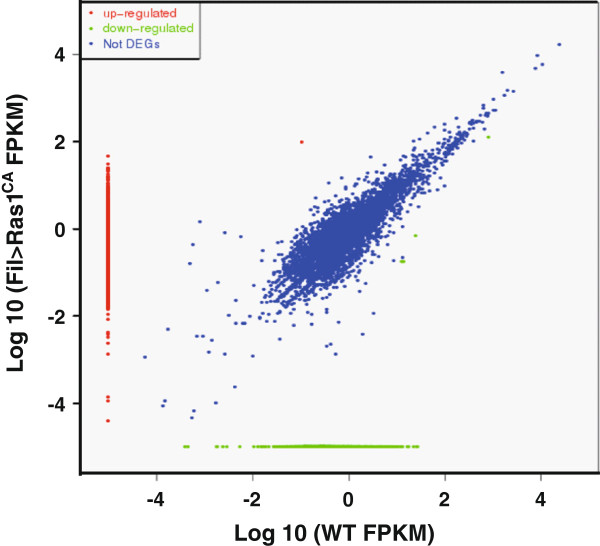
**Gene expression in** ***Ras1*** ^**CA**^ **-overexpressed and WT PSGs.** All reads were aligned to predicted genes and are shown as log10 values derived from cDNAs of the *Ras1* ^CA^-overexpressed and WT PSGs (F-test, P, <2.2 × 10^-16^). The Ras1-upregulated genes and downregulated genes are marked with red and green spots, respectively.

Importantly, RNA-Seq revealed 2,636 DEGs, with 1708 upregulated and 938 downregulated genes in the *Ras1*^CA^-overexpressed PSG compared to the WT PSG (Figure 1). Next, the DEGs were subjected to functional annotation and qPCR verification.

### Functional annotation of DEGs

**Figure 2:**
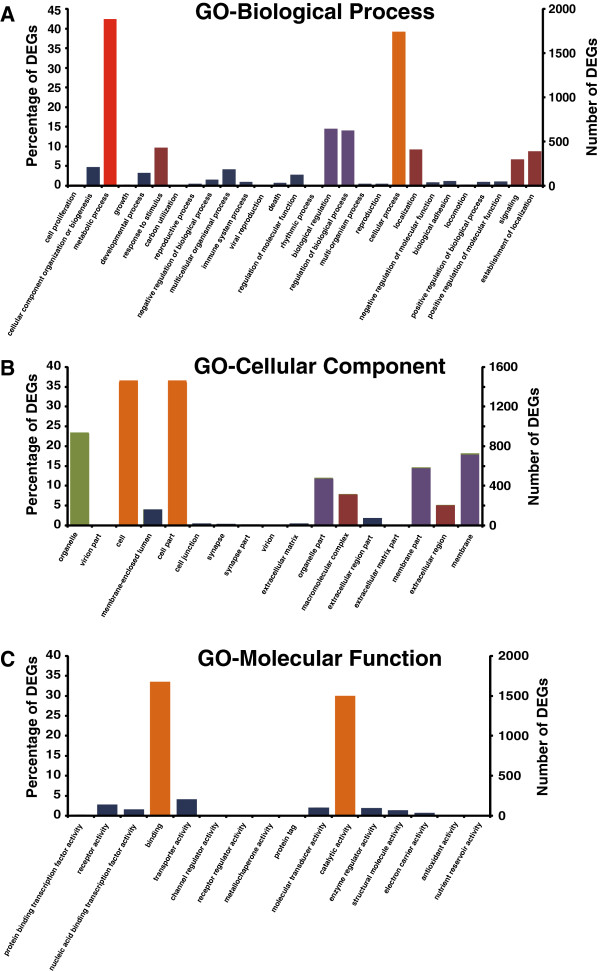
**Histogram presentation of Gene Ontology classification of DEGs in** ***Ras1*** ^**CA**^ **-overexpressed and WT PSGs.** The results are summarized in three main categories: biological process **(A)**, cellular component **(B)**, and molecular function **(C)**. The right *y*-axis indicates the number of genes in a category. The left *y*-axis indicates percentage of DEGs in a specific category. Different color represents different percentage: dark blue, 0-5%; dark red, 5-10%, dark purple, 10-20%; green, 20-30%; orange, 30-40%; red, over 40%.

Gene ontology (GO) assignments were used to classify the functions of DEGs revealed by transcriptomic analysis. The DEGs were termed by GO ontology in three categories, namely biological process, cellular component, and molecular function. In total, 2512 DEGs (95.3% of 2636) were annotated in 60 GO functional groups (Figure 2). In the category of biological process, over 43% of DEGs were distributed in “metabolic process”, nearly 40% DEGs were classified into “cellular process”, while very few DEGs were found in “growth”, “carbon utilization”, “viral reproduction”, “rhythmic process”, and “locomotion” (Figure 2A). In the category of cellular component, over 35% of DEGs were distributed in “cell” and “cell part”, however, very small numbers of DEGs were found in “virion part”, “synapse”, and “extracellular matrix part” (Figure 2B). In the category of molecular function, the terms “binding” and “catalytic activity” enriched 32% and 30% of DEGs, respectively. By contrast, few DEGs were distributed in “channel regulator activity”, “receptor regulator activity”, and “metallochaperone activity” (Figure 2C).

To identify the biological pathways that are active in the *Ras1*^CA^-overexpressed PSG, we mapped the DEGs to the reference canonical pathways in Kyoto Encyclopedia of Genes and Genomes (KEGG) [22]. Among the 2636 DEGs, 1,941 sequences predicted to encode enzymes with enzyme commission (EC) numbers were mapped into 277 KEGG pathways. The top 25 KEGG pathways are shown in Table 3. The top 5 KEGG pathways with most representations by the DEGs were “purine metabolism”, “spliceosome”, “pathways in cancer”, “RNA transport”, and “HTLV-1 infection”, implying that these diverse metabolic processes are active in the *Ras1*^CA^-overexpressed PSG. “Purine metabolism” is essential, providing components of the nucleotides, DNA and RNA, and the energy currency of the cell, ATP [23]. In parallel, “Pyrimidine metabolism” also ranks on the top 6 KEGG pathway (Table 3). In the *Ras1*^CA^-overexpressed PSG, the DNA content is nearly doubled and endoreplication is enhanced in comparison with the WT PSG, indicating that more active nucleotide metabolism is required [2]. “Spliceosome” is complex molecular machinery involved in removal of introns from mRNA precursors [24]. A lot of DEGs in this pathway suggest occurrence of high frequency of mRNA splicing for translation in the *Ras1*^CA^-overexpressed PSG. Interestingly, a large number of DEGs are found distributed in “pathways in cancer”. As an important oncogene, *Ras* plays important roles in both normal development and aberrant biological processes, such as tumorigenesis and developmental disorders [25]. It is likely that, apart from the phosphorylational regulation, Ras could also activate “pathways in cancer” at the transcriptional level. “RNA transport” from the nucleus to the cytoplasm is fundamental for gene expression regulation. Most eukaryotic RNAs are produced in the nucleus by RNA polymerase I, II, or III. The RNA molecules undergo a variety of posttranscriptional processing events, and a majority of them are localized to their functional sites in the cell [26]. In accordance with this result, ribosome biogenesis for mRNA translation was stimulated in the *Ras1*^CA^-overexpressed PSG [2]. Unexpectedly, some DEGs were annotated into “HTLV-1 infection pathway”, but the silkworm cannot be affected by this human virus. Our preliminary data shows that virus-resistance is enhanced when *Ras1*^CA^ is globally overexpressed in the transgenic silkworm, Actin3-GAL4 > UAS-Ras1^CA^, suggesting that the DEGs in “HTLV-1 infection pathway” might play a role in antivirus in *Bombyx*.

**Table 3:** **A comparison between qPCR verification results and transcriptomic data of DEGs in five KEGG pathways**

Pathway	Number of DEGs verified by qPCR	Number of matched DEGs	Ratio of matched DEGs	Number of unmatched DEGs	Ratio of unmatched DEGs
**Pathways in cancer**	22	18	81.8%	4	18.2%
**Insulin signaling**	18	16	88.9%	2	11.1%
**MAPK signaling**	13	11	84.6%	2	15.4%
**Purine metabolism**	15	12	80.0%	3	20.0%
**Pyrimidine metabolism**	11	8	72.7%	3	27.3%

In short, these annotations provide a valuable insight into the specific processes, functions, and pathways and facilitate the identification of DEGs resulted from the *Ras1*^CA^-overexpressed PSG. Next, the DEGs in several important and top KEGG pathways were chosen for qPCR verification (Table 3).

### qPCR verification of DEGs in “pathways in cancer”, “insulin signaling pathway”, and “MAPK signaling pathway”

In our previous study, we have determined that *Ras1*^CA^ overexpression in the PSG increases Ras activity, resulting in phosphorylation of the Ras downstream effector proteins, Raf and PI3K110, which in turn activate the Raf-MAPK and PI3K-TORC1 pathways, respectively [2]. Surprisingly, among all the 277 KEGG pathways, a large number of DEGs are distributed in “pathways in cancer”, “insulin signaling”, and “MAPK signaling pathway” (Additional file 1: Figure S1, Additional file 2: Figure S2 and Additional file 3: Figure S3), which rank on the top 3, 9, and 15 KEGG pathways containing 49, 35, and 32 DEGs, respectively (Table 4).

**Table 4:** **Number of KEGG orthologs (KO) in pathways with top mapped KOs**

Pathway	Pathway type	DEGs
**ko00230, Purine metabolism**	**Metabolism/Nucleotide metabolism**	**67**
ko03040, Spliceosome	Genetic information processing/Transcription	52
**ko05200, Pathways in cancer**	**Human diseases/Cancers**	**49**
ko03013, RNA transport	Genetic information processing/Translation	47
ko05166, HTLV-I infection	Human diseases/Infectious diseases: Viral	44
**ko00240, Pyrimidine metabolism**	**Metabolism/Nucleotide Metabolism**	**42**
ko04110, Cell cycle	Cellular processes/Cell growth and death	42
ko04510, Focal adhesion	Cellular processes/Cell communication	38
**ko04910, Insulin signaling pathway**	**Organismal systems/Endocrine system**	**35**
ko03008, Ribosome biogenesis in eukaryotes	Genetic information processing/Translation	35
ko05016, Huntington's disease	Human diseases/Neurodegenerative diseases	34
ko04146, Peroxisome	Cellular processes/Transport and catabolism	34
ko00561, Glycerolipid metabolism	Metabolism/Lipid metabolism	32
ko05010, Alzheimer's disease	Human diseases/Neurodegenerative diseases	32
**ko04010, MAPK signaling pathway**	**Environmental information processing /Signal transduction**	**32**
ko04111, Cell cycle - yeast	Cellular processes/Cell growth and death	32
ko04144, Endocytosis	Cellular processes/Transport and catabolism	31
ko04810, Regulation of actin cytoskeleton	Cellular processes/Cell motility	30
ko00564, Glycerophospholipid metabolism	Metabolism/Lipid metabolism	29
ko05169, Epstein-Barr virus infection	Human diseases/Infectious diseases: Viral	29
ko00260, Glycine, serine and threonine metabolism	Metabolism/Amino acid metabolism	28
ko04120, Ubiquitin mediated proteolysis	Genetic information processing/Folding, sorting and degradation	28
ko04113, Meiosis - yeast	Cellular processes/Cell growth and death	28
ko03010, Ribosome	Genetic information processing/Translation	25
ko04145, Phagosome	Cellular processes/Transport and catabolism	25

The datas marked with boldface were chosen for further analysis.

**Figure 3:**
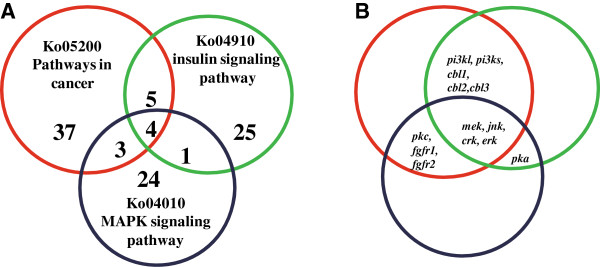
**DEGs distribution in “pathways in cancer”, “insulin signaling pathway” and” MAPK signaling pathway”. (A)** Showing the number of DEGs. **(B)** Showing the common DEGs.

**Figure 4:**
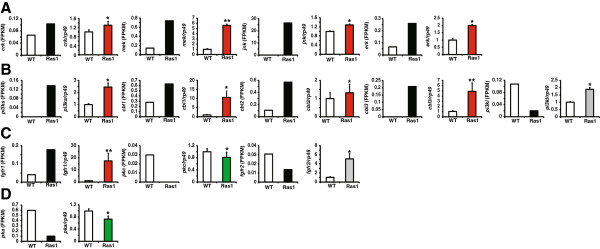
**Verification of transcriptomic results of DEGs in “pathways in cancer”, “insulin signaling pathway”, and “MAPK signaling pathway” by qPCR. (A)** Common DEGs in all the three pathways. **(B)** Common DEGs in “pathways in cancer” and “insulin signaling pathway”. **(C)** Common DEGs in “pathways in cancer” and “MAPK signaling pathway”. **(D)** Common DEG in “insulin signaling pathway” and “MAPK signaling pathway”. The transcriptional results of DEGs (FPKM) are marked with black. The q-PCR data of DEGs upregulated and downregulated by Ras1 are marked with red and green. The mis-matched DEGs are marked with gray. WT: wildtype; Ras1: Fil-GAL4 > UAS-Ras1^CA^.

Since these three KEGG pathways are highly related to each other, we analyzed their common DEGs and verified them by qPCR (Figure 3). First, 4 DEGs were found in all the three pathways, including *crk*, *mek*, *jnk*, and *erk*. qPCR data were in agreement with the transcriptomic results, showing that all these 4 genes are upregulated in the *Ras1*^*CA*^-overexpressed PSG (Figure 4A). Second, 5 DEGs were observed in both “pathways in cancer” and “insulin signaling pathway”, including *pi3kl*, *pi3ks*, *cbl1*, *cbl2*, and *cbl3* (Figure 4B). The transcriptomic results and qPCR data showed that *pi3ks*, *cbl1*, *cbl2*, and *cbl3* are all upregulated by Ras1^CA^, whereas the qPCR data of *pi3kl* did not match its transcriptomic result suggesting it might also be upregulated by Ras1^CA^. Third, there were 3 DEGs in both “pathways in cancer” and “MAPK signaling pathway”, including *fgfr1*, *pkc*, and *fgfr2*. The transcriptomic results and qPCR data showed the *fgfr1* and *pkc* are upregulated and downregulated by Ras1^CA^, respectively, while *fgfr2* might be downregulated by Ras1^CA^ (Figure 4C). Finally, *pka*, a single DEG in both “insulin signaling pathway” and “MAPK signaling pathway”, was downregulated by Ras1^CA^ (Figure 4D).

**Figure 5:**
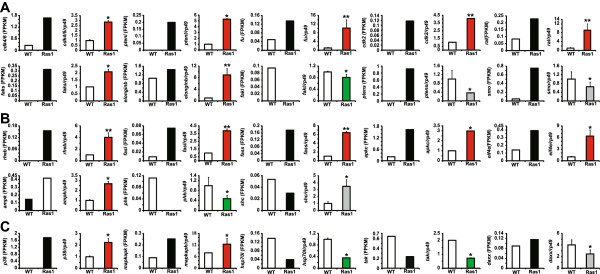
**Verification of transcriptomic results of DEGs only in “pathways in cancer”, “insulin signaling pathway”, or “MAPK signaling pathway” by qPCR.** DEGs only in “pathways in cancer” **(A)**, “insulin signaling pathway” **(B)**, “MAPK signaling pathway” **(C)**. The transcriptional results of DEGs (FPKM) are marked with black. The q-PCR data of DEGs upregulated and downregulated by Ras1 are marked with red and green. The mis-matched DEGs are marked with gray. WT: wildtype; Ras1: Fil-GAL4 > UAS-Ras1^CA^.

We randomly selected some DEGs in each individual pathway for qPCR verification (Figure 5). In “pathways in cancer”, both the transcriptomic results and qPCR data revealed that 7 DEGs, including *cdk4/6*, *ptenl*, *fu*, *cdk2*, *ral*, *faks*, and *elongb*, are all upregulated by Ras1^CA^, while *fakl* is downregulated. Although the transcriptomic results suggested that *ptens* and *smo* are upregulated by Ras1^CA^, qPCR data showed that they might be downregulated (Figure 5A). In “insulin signaling pathway”, 7 DEGs, including *rheb*, *fasl*, *fass*, *apkc*, *apkc*, *eif4e*, and *ampk*, are all upregulated by Ras1^CA^, while *phk* is downregulated, and *shc* might be upregulated (Figure 5B). In “MAPK signaling pathway”, *p38* and *mapkapk* are upregulated by Ras1^CA^, while *hsp70s* and *tak* are downregulated, and *daxx* might be downregulated (Figure 5C).

### qPCR verification of DEGs in “purine metabolism” and “pyrimidine metabolism”

Interestingly, abundant DEGs were annotated in two major nucleotide metabolism pathways, “purine metabolism” and “pyrimidine metabolism” (Additional file 4: Figure S4 and Additional file 5: Figure S5), ranking on the top 1 and 6 KEGG pathways which contain 67 and 42 DEGs, respectively (Table 3).

**Figure 6:**
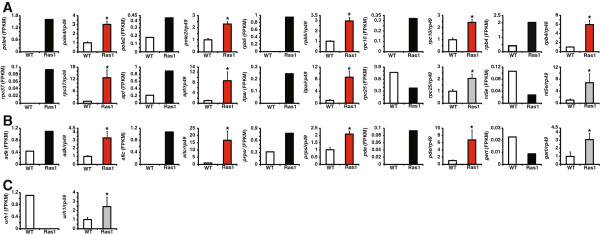
**Verification of transcriptomic results of DEGs in “purine metabolism” and “pyrimidine metabolism” by qPCR. (A)** Common DEGs in both pathways. **(B)** DEGs only in “purine metabolism”. **(C)** DEGs only in “pyrimidine metabolism”. The transcriptional results of DEGs (FPKM) are marked with black. The q-PCR data of DEGs upregulated and downregulated by Ras1 are marked with red and green. The mis-matched DEGs are marked with gray. WT: wildtype; Ras1: Fil-GAL4 > UAS-Ras1^CA^.

We thus verified the above-mentioned hypothesis that Ras might activate nucleotide metabolism by qPCR verification of some randomly selected DEGs in both “purine metabolism” and “pyrimidine metabolism” (Figure 6). We first analyzed 10 of the 28 common DEGs in both pathways, including *pole4*, *pole2*, *rpb5*, *rpc10*, *rpb4*, *rpc37*, *apf*, *itpa*, *rpc25*, and *nt5e*. Different from the transcriptomic results, qPCR data suggest that *rpc25* and *nt5e* might be upregulated rather than downregulated by Ras1 (Figure 6A). We then analyzed 5 of the 39 DEGs only in “purine metabolism”, including *adk*, *allc*, *prps*, *pde*, and *gart*. Although *gart* expression was inconsistent between its transcriptomic and qPCR data, all the other DEGs are upregulated (Figure 6B). In addition, *urh1*, one of DEGs only in “pyrimidine metabolism” is also upregulated (Figure 6C). Taken together, Ras1^CA^ overexpression in the PSG upregulates most, if not all, DEGs in “purine metabolism” and “pyrimine metabolism”.

### Identification of Ras1 downstream signals in regulating DEGs by small-molecule inhibitor treatments

We next investigated which downstream pathway(s) that Ras1 utilizes to regulate DEGs in the *Bombyx* PSG by injecting small-molecule inhibitors of the Ras downstream effectors (Rafi, Raf inhibitor; LY294002, PI3K inhibitor; rapamycin, TORC1 inhibitor; 15 μg/larva) into the *Ras1*^CA^-overexpressed silkworm larvae [10]. Some *Ras1*^CA^-upregulated DEGs, which are consistent in both transcriptomic results and qPCR data, were chosen for inhibitor treatment experiments by qPCR analysis to examine their expression levels.

**Figure 7:**
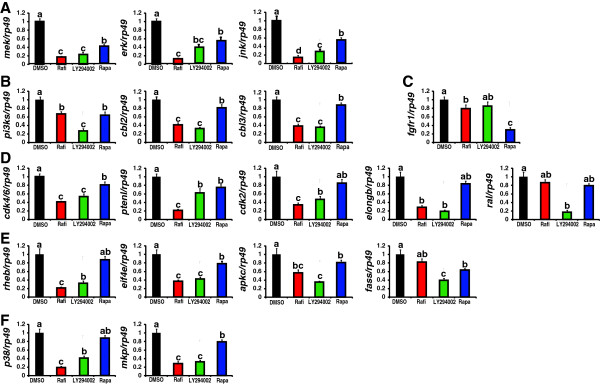
**Small-molecule chemical inhibitor treatments to identify which Ras1 downstream signaling pathways are involved in regulating DEGs in “pathways in cancer”, “insulin signaling pathway”, and “MAPK signaling pathway”. (A)** Common DEGs in all the three pathways. **(B)** Common DEGs in “pathways in cancer” and “insulin signaling pathway”. **(C)** A common DEG in “pathways in cancer” and “MAPK signaling pathway”. **(D)** DEGs only in “pathways in cancer”. **(E)** DEGs only in “insulin signaling pathway”. **(F)** DEGs only in “MAPK signaling pathway”. Small-molecule chemical inhibitors of the Ras downstream effectors (Rafi, Raf inhibitor; LY294002, PI3K inhibitor; Rapa, rapamycin, TORC1 inhibitor; 15 μg/larva) are injected into *Ras1* ^CA^-overexpressed silkworm larvae. Inhibitors are marked with different color: black, DMSO as a control; red: Raf inhibitor; green, LY294002; blue: rapamycin.

First, we detected the common DEGs annotated in “pathways in cancer”, “insulin signaling pathway”, and “MAPK signaling pathway” by qPCR (Figure 7A-C). The mRNA levels of *mek*, *erk*, and *jnk* distributed in all the three pathways were decreased to 10-20% by Rafi and 20-40% by LY294002, whereas rapamycin treatment showed weaker inhibitory effects (40-60%) (Figure 7A). For *pi3ks*, *cbl2*, and *cbl3*, the three DEGs presented in both “pathways in cancer” and “insulin signaling pathway”, LY294002 and rapamycin showed the strongest and weakest inhibitory effects, respectively (Figure 7B). By contrast, rapamycin strongly inhibited expression of *fgfr1*, the DEGs distributed in “pathways in cancer” and “MAPK signaling” (Figure 7C).

Second, we detected the individual DEGs annotated in “pathways in cancer” (Figure 7D), “insulin signaling pathway” (Figure 7E), and “MAPK signaling pathway” (Figure 7F) by qPCR. For most of the DEGs, Raf inhibitor exhibited the strongest inhibitory effects, while rapamycin showed little to no inhibitory effects.

**Figure 8:**
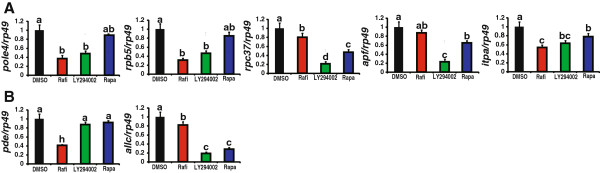
**Small-molecule inhibitor treatments to identify which Ras1 downstream signaling pathways are involved in regulating DEGs in “purine metabolism” and “pyrimidine metabolism”. (A)** Common DEGs in both pathways. **(B)** DEGs only in “purine metabolism”. Small-molecule chemical inhibitors of the Ras downstream effectors (Rafi, Raf inhibitor; LY294002, PI3K inhibitor; Rapa, rapamycin, TORC1 inhibitor; 15 μg/larva) are injected into *Ras1* ^CA^-overexpressed silkworm larvae. Inhibitors are marked with different color: black, DMSO as a control; red: Raf inhibitor; green, LY294002; blue: rapamycin.

Third, we detected the DEGs annotated in “purine metabolism” and “pyrimidine metabolism” (Figure 8). For DEGs in both pathways, LY294002 exhibited the strongest inhibitory effects (Figure 8A). For the two DEGs only in “purine metabolism”, Raf inhibitor and LY294002 showed the strongest inhibitory effects on *pde* and *allc*, respectively (Figure 8B).

In summary, inhibitors of the Ras downstream effectors showed inhibitory efforts on different DEGs to varying degrees indicating that both Raf-MAPK and PI3K-TORC1 pathways are involved in the transcriptional regulation of those DEGs. Interestingly, similar results were observed in mammalian cells in which *Ras* is overexpressed or transformed [27, 28].

## Discussion

### Ras1 transcriptionally activates its downstream Raf-MAPK and PI3K-TORC1 pathways

On a genome-wide scale, the identification of Ras-responsive genes has become feasible using different transcriptomic tools. For example, subtractive suppression hybridization was performed in immortalized, non-tumorigenic rat embryo fibroblasts and in Ras-transformed cells. The results have shown that many DEGs are involved in almost all aspects of cellular growth control and cell survival [29]. A microarray was conducted in *Ras*^CA^-transformed mouse embryonic fibroblasts, showing that many genes encoding cell growth-related proteins are upregulated [30]. The results of Ras-induced gene expression profiling studies based on subtractive suppression hybridization and microarrays were extensively summarized [27], revealing that Kruppel-like factor 5, the CD44 antigen, and members of the epidermal growth factor (EGR)-family are common Ras downstream effectors [28]. Using Illumina-Solexa sequencing to analyze DEGs in the PSG, here we found that many Ras1-induced genes are distributed in “pathways in cancer”, “insulin signaling”, and “MAPK signaling pathway”. The transcriptomic analysis illustrates that, apart from phosphorylational regulation, Ras1 can also activate its downstream Raf-MAPK and PI3K-TORC1 pathways at the transcriptional level (Figure 3, 4 and 5, Additional file 1: Figure S1, Additional file 2: Figure S2 and Additional 3: Figure S3, Table 4). To our knowledge, this is the first report that Ras1 can transcriptionally activate its downstream pathways at a global level.

### Ras1 transcriptionally activates genes involved in nucleotide metabolism and cell cycle for increasing DNA content and inducing endoreplication

Earlier studies have shown that a lot of key enzymes of nucleotide metabolism and DNA biosynthesis, such as CTP synthetase, thymidylate synthase, dihydrofolate reductase, IMP dehydrogenase, ribonucleotide reductase, DNA polymerase, and DNA methyltransferase, are markedly upregulated in certain tumor cells, which supports the excessive proliferation of transformed cells [31]. The microarray conducted in *Ras*^CA^-transformed mouse embryonic fibroblasts revealed that many genes encoding DNA-associated proteins (involved in DNA replication and DNA repair) are upregulated as well [30]. Interestingly, a microarray analysis of *Ras*-overexpressed hemocytes in the fruitfly, *Drosophila melanogaster*, showed that a large number of genes that are functionally important in cell cycle regulation and DNA replication were upregulated [32]. We have previously shown that compared to the WT PSG, total DNA content is nearly doubled in the *Ras1*^CA^-overexpressed PSG [2]. Moreover, in comparison with the WT PSG, BrdU incorporation in the *Ras1*^CA^-overexpressed PSG is much higher indicating enhanced endoreplicative cycles [2]. In this study, we found many Ras1-induced genes are enriched in “purine metabolism”, “pyrimidine metabolism”, and “cell cycle”, which ranks top 1, 6, and 7, respectively (Figure 6, Table 3). Therefore, it is likely that Ras1 transcriptionally activates genes involved in nucleotide metabolism and cell cycle for increasing DNA content and inducing endoreplication in the PSG.

## Conclusion

About 46 years before, it has been hypothesized that fibroin production in the *Bombyx* PSG is directly proportional to silk yield and determined by its gland size and protein synthesis [1]. Based on this hypothesis, we have generated a transgenic silkworm, Fil > Ras1^CA^, for improving silk yield. Importantly, overexpression of *Ras1*^CA^ increases gland size and protein synthesis in the PSG, improving fibroin production and silk yield by 60%. At the molecular level, we have determined that Ras activation enhances phosphorylation levels of Ras downstream effector proteins, Raf and PI3K110, and thus activates its downstream Raf-MAPK and PI3K-TORC1 pathways [2]. To better understand the molecular mechanisms how *Ras1*^CA^ overexpression in the PSG improves fibroin production and silk yield, we performed both proteomics and transcriptomics. Unfortunately, in spite of the discovery that *Ras1*^CA^ upregulates *bcpi* to inhibit cathepsin activity and thus to prevent PSG destruction, we were not able to better understand how *Ras1*^CA^ improves fibroin production and silk yield using proteomics [10].

**Figure 9:**
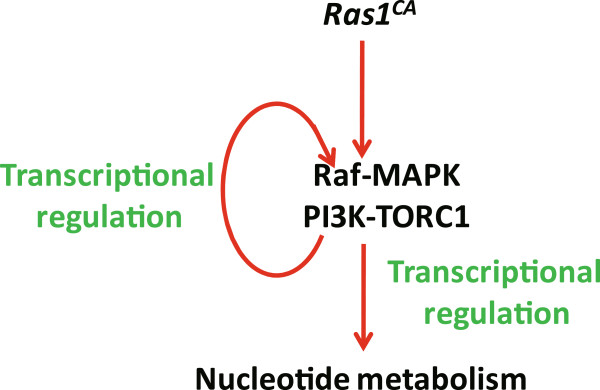
**A proposed model shows that Ras1** ^**CA**^ **activates its downstream Raf-MAPK and PI3K-TORC1 pathways at both phosphorylational and transcriptional levels and upregulates genes in “nucleotide metabolism” for inducing endoreplication.**

The transcriptomic results of the Ras1^CA^-overexpressed PSG presented here underlie a wide array of DEGs in many KEGG pathways. Importantly, we have discovered that a large number of DEGs in “pathways in cancer”, “insulin signaling”, and “MAPK signaling pathway” are upregulated by *Ras1*^CA^ overexpression in the PSG. Combined with our previous findings [2] and the present studies (Figure 9), we conclude that Ras1 activates its downstream Raf-MAPK and PI3K-TORC1 pathways at both phosphorylational and transcriptional levels. Moreover, we find that Ras1 upregulates genes in “nucleotide metabolism” and “cell cycle” for increasing DNA content and inducing endoreplication (Figure 9). This study has advanced our knowledge on how *Ras1*^*CA*^ overexpression in the PSG improves fibroin production and silk production.

## Methods

### Animals

The *Bombyx* strain, Dazao, was reared on fresh mulberry leaves in the laboratory at 25°C under 14 h light/10 h dark cycles. The transgenic silkworm Fil > Ras1^CA^ was obtained as described previously by crossing Fil-GAL4 with UAS-Ras1^CA^[2]. The transgenic silkworms were reared under the same condition as the WT silkworms.

### Dissection of the PSGs

The silkworm PSGs were dissected from the *Ras1*^CA^-overexpressed and WT silkworms at the early wandering stage, when silkworms just begin to spin [2, 10]. The PSGs were used for Illumina-Solexa sequencing and qPCR. Throughout the paper, all qPCR experiments were performed in 3 biological duplicates.

### RNA extraction

For Illumina sequencing, total RNA from the *Ras1*^CA^-overexpressed PSG or the WT PSG was isolated with TRIzol (Invitrogen, Carlsbad, CA, USA). To remove any residual DNA, samples were pretreated with RNase-free DNase I (New England BioLabs, Ipswich, MA, USA) for 30 minutes at 37°C. RNA quality was first verified using a 2100 Bioanalyzer RNA Nanochip (Agilent, Santa Clara, CA) with RNA Integrity Number (RIN) value over 8.5. RNA was then quantified using NanoDrop ND-1000 Spectrophotometer (NanoDrop, Wilmington, DE).

### Library preparation and Illumina sequencing

The RNA-seq libraries were prepared using Illumina’skit following the manufacturer’s protocol (Illumina, San Diego, CA). Approximately 20 μg of total RNA from the *Ras1*^CA^-overexpressed PSG or the WT PSG was used to isolate mRNA using Sera-mag Magnetic Oligo (dT) Beads (Illumina). To avoid cDNA synthesizing bias by priming, the purified mRNA was fragmented into small pieces (100–400 bp) using divalent cations at 94°C for 5 minutes. The double-stranded cDNA was first synthesized using the SuperScript Double-Stranded cDNA Synthesis kit (Invitrogen, Camarillo, CA) with random hexamer (N6) primers (Illumina). Then the synthesized cDNA was subjected to end-repair and phosphorylation using T4 DNA polymerase, Klenow DNA polymerase, and T4 PNK. These repaired cDNA fragments were 3' adenylated using Klenow Exo- (3' to 5' exo minus, Illumina).Illumina paired-end adapters were ligated to the ends of these 3'-adenylated cDNA fragments.The ligated cDNA was then enriched with 15 rounds of PCR amplification using PCR Primer PE 1.0 and PCR Primer PE 2.0 (Illumina) with Phusion DNA Polymerase.The libraries were sequenced using Illumina Highseq 2000 platform according to the manufacturer’s instructions. Illumina sequencing was performed at Suzhou Encode Genomics Biotechnology Co-Ltd (Encode Genomics; Suzhou, China).

### Raw data preprocess

Preprocessing was carried out with a stringent filtering process. First, we removed reads that do not pass the built-in Illumina's software Failed-Chastity filter according to the relation "failed-chastity < = 1", using a chastity threshold of 0.6, on the first 25 cycles. Second, we discarded all reads with adaptor contamination. Third, we ruled out low-quality reads containing ambiguous sequences "N". Finally, the reads with more than 10% Q < 20 bases were also removed.

### Genome mapping and abundance analysis

Quality-filtered reads were then aligned to the *Bombyx* genome (release_2.0, ftp://silkdb.org/pub/current/Genome/ by TopHat (version 2.0.4) [20] with the parameters “--bowtie1 -r 0 --mate-std-dev 50 -N 3--solexa1.3-quals” (insert size is set as 0). The resulting alignment data from Tophat were then fed to an assembler Cufflinks (version 2.0.1) to assemble aligned RNA-Seq reads into silkworm genome database (silkworm_glean.gff) and silkworm gene database (silkgenome.fa). Unigene abundances were measured by Fragments per kb of exon per million fragments mapped (FPKM) using the formula FPKM = (1,000,000*C)/ (N*L*1,000)[21].

### Functional annotations

The DEGs in *Ras1*^CA^-overexpressed and WT PSGs were functional annotated by GO annotation and KEGG annotation. For GO annotation, the DEGs were first blasted against uniprot knowledgebase (including Swiss-Prot and TrEMBL) (UniProtKB; http://www.uniprot.org) to get uniprot IDs. Then the uniprot IDs were assigned to GO terms at three basic catergories including molecular function, biological process, and cellular component. For KEGG annotation, DEGs were functionally annotated with KAAS (KEGG Automatic Annotation Server) by BLAST comparisons against the manually curated KEGG GENES database. The result contains KO (KEGG Orthology) assignments and automatically generated KEGG pathways.

### qPCR

Total RNA of the *Ras1*^CA^-overexpressed PSG or the WT PSG was extracted using TRIzol (Invitrogen). qPCR was performed as previously described [2, 33]. The primers used in this paper are listed in Additional file 6: Table S1 (Supporting Information).

### Chemical inhibitor treatment

Small-molecule chemical inhibitors of the Ras downstream effectors (Rafi, Raf inhibitor, Santa Cruz; LY294002, PI3K inhibitor, Sigma; rapamycin, TORC1 inhibitor, Sigma; 15 μg/larva) were injected into the Fil > Ras1^CA^ larvae at the EW stage. At 24 h after the first injection, the PSGs were dissected for qPCR as previously described [10].

### Statistics

Experimental data were analyzed with the Student’s *t*-test and ANOVA. *t*-test: *, *p* < 0.05; **, *p* < 0.01. ANOVA: the bars labeled with different lowercase letters are significantly different (*p* < 0.05). Throughout the paper, values are represented as the mean ± standard deviation of at least 3 independent experiments.

### Availability of supporting data

The RNA-seq raw data were deposited to NCBI SRA with the accession number SRP026709 (http://www.ncbi.nlm.nih.gov/sra/?term=SRP026709).

## Electronic supplementary material

Additional file 1: Figure S1: DEGs distributed in “pathways in cancer” (KEGG) signaling network. DEGs are marked with red square. (EPS 2 MB)

Additional file 2: Figure S2: DEGs distributed in “insulin signaling pathway” (KEGG) signaling network. DEGs are marked with red square. (EPS 1 MB)

Additional file 3: Figure S3: DEGs distributed in “MAPK signaling pathway” (KEGG) signaling network. DEGs are marked with red square. (EPS 2 MB)

Additional file 4: Figure S4: DEGs distributed in “purine metabolism” (KEGG) signaling network. DEGs are marked with red square. (EPS 7 MB)

Additional file 5: Figure S5: ADEGs distributed in “pyrimidine metabolism” (KEGG) signaling network. DEGs are marked with red square. (EPS 8 MB)

Additional file 6: Table S1: List of primers used for qPCR verification. (DOCX 23 KB)
