# Supplementary material for: Transcriptomic analysis of differentially expressed genes in the Ras1CA-overexpressed and wildtype posterior silk glands
Source: BMC Genomics. 2014 Mar 9;15(1):182. doi: 10.1186/1471-2164-15-182 (PMC4029079; doi:10.1186/1471-2164-15-182)
Supplement: Supplementary file 6 — Additional file 6: Table S1: List of primers used for qPCR verification. (DOCX 23 KB) [file 12864_2013_7023_MOESM6_ESM.docx]

**Table S1. List of primers used for q-PCR verification.**

| **Pathway distribution** | **Gene name** | **Primer sequence** |
| --- | --- | --- |
| Pathways in cancer  Insulin signaling pathway  MAPK signaling pathway | *crk* | forward 5’-gactgagagtggcgtgtttc-3’ |
|  |  | reverse 5’-tctagggtctggttctgctg-3’ |
|  | *jnk* | forward 5’-accagaacctcaaacccatc-3’ |
|  |  | reverse 5’-ggtttgaggttctggtagcg-3’ |
|  | *mek* | forward 5’-tccatctggcatcttctctg-3’ |
|  |  | reverse 5’-gtggaaggcattaggtccat-3’ |
|  | *erk* | forward 5’-caagctgttgaagacccaga-3’ |
|  |  | reverse 5’-cgaagtcgcaaatcttcaaa-3’ |
| Pathways in cancer  Insulin signaling pathway | *pi3kl* | forward 5’-tgaacgcgtacaactgcata-3’ |
|  |  | reverse 5’-tgtcggtgtactttcgaagc-3’ |
|  | *pi3ks* | forward 5’-cgagacactcgtgatggaac-3’ |
|  |  | reverse 5’-ctgttacatgttgccaggga-3’ |
|  | *cbl1* | forward 5’-aggaaatgcaagcaggctat-3’ |
|  |  | reverse 5’-tcttcgtgatcctgaactgg-3’ |
|  | *cbl2* | forward 5’-cggtgagatcctacagacga-3’ |
|  |  | reverse 5’-gcatatcttgcaaagctgga-3’ |
|  | *cbl3* | forward 5’-cctctaacccatgacaagca-3’ |
|  |  | reverse 5’-attggtcgctttagatccga-3’ |
| Pathways in cancer  MAPK signaling pathway | *pkc* | forward 5’-cgctgatgttatccgagcta-3’ |
|  |  | reverse 5’-aacgtcatcgtcctgaatga-3’ |
|  | *fgfr1* | forward 5’-tatggacagcatggacacct-3’ |
|  |  | reverse 5’-gcgatttgacgacagaaaga-3’ |
|  | *fgfr2* | forward 5’-actcgatgatgatgtcggaa-3’ |
|  |  | reverse 5’-acaccaaagccatcatttca-3’ |
| Insulin signaling pathway  MAPK signaling pathway | *pka* | forward 5’-tgaacgatttcgaacggata-3’ |
|  |  | reverse 5’-aggcacatactccaacacca-3’ |
| Pathways in cancer | *cdk4/6* | forward 5’-aatggagggcagattgtagc-3’ |
|  |  | reverse 5’-ttccacatgttcaaacacca-3’ |
|  | *ptenl* | forward 5’-gatgaagatgcgcaaagaga-3’ |
|  |  | reverse 5’-ggctgtaatgtttgtgttgctt-3’ |
|  | *fu* | forward 5’-ccactagcgcattcagtgtt-3’ |
|  |  | reverse 5’-tttgactgcatccgctttag-3’ |
|  | *cdk2* | forward 5’-gatctgacgaagggaccact-3’ |
|  |  | reverse 5’-acggcggttgagtagaactt-3’ |
|  | *ral* | forward 5’-gctgcataaggtgatcatgg-3’ |
|  |  | reverse 5’-caggctcagtgatggagaaa-3’ |
|  | *faks* | forward 5’-ccgtcaccaccagtcagata-3’ |
|  |  | reverse 5’-actcgactcctcaccgtctc-3’ |
| Pathways in cancer | *elonginb* | forward 5’-acctcctccaagtcaaatgc-3’ |
|  |  | reverse 5’-cagtgagcctttgctgtgtt-3’ |
|  | *fakl* | forward 5’-ggagactctaccggagaacg-3’ |
|  |  | reverse 5’-acacagattgtcgcagctct-3’ |
|  | *ptens* | forward 5’-gacatacgacccgagcaa-3’ |
|  |  | reverse 5’-cgaacggtagtagcacgaa-3’ |
|  | *smo* | forward 5’-ccaaagatggcactagacga-3’ |
|  |  | reverse 5’-aggtggaaatacgcagcttt-3’ |
| Insulin signaling pathway | *rheb* | forward 5’-ggaaggccaatttgttgatt-3’ |
|  |  | reverse 5’-tgccaaccatgtccagtaat-3’ |
|  | *fasl* | forward 5’-caacatactgggcatcaagg-3’ |
|  |  | reverse 5’-cgtcgtaccctcgctctaat-3’ |
|  | *fass* | forward 5’-tggtgaacgggactaatgg-3’ |
|  |  | reverse 5’-acccatcgtctgctgtca-3’ |
|  | *apkc* | forward 5’-tgaaatgggtggatgaagaa-3’ |
|  |  | reverse 5’-ctgtactcgagtgcctggaa-3’ |
|  | *eif4e* | forward 5’-ggaggatccgattaacgaaa-3’ |
|  |  | reverse 5’-tttgtcttcaccacgtgtcc-3’ |
|  | *ampk* | forward 5’-atggagaatggaggcatgat-3’ |
|  |  | reverse 5’-ttggtggaccagacactttc-3’ |
|  | *phk* | forward 5’-ttcctccagatgatgtccaa-3’ |
|  |  | reverse 5’-tatcgtcgacctcttcttcg-3’ |
|  | *shc* | forward 5’-ccaaggaatgcatagcaaga-3’ |
|  |  | reverse 5’-gatactctgggcatgtcgtg-3’ |
| MAPK signaling pathway | *p38* | forward 5’-ggctattagtggccctcttg-3’ |
|  |  | reverse 5’-ccaatattaagacctgcccaa-3’ |
|  | *mapkapk* | forward 5’-agacaggccgcagagataat-3’ |
|  |  | reverse 5’-ccttagcgaaaccaaagtcc-3’ |
|  | *hsp70l* | forward 5’-aggaaccagctcgaaaccta-3’ |
|  |  | reverse 5’-tataacggaccactgcctca-3’ |
|  | *tak1* | forward 5’-acaaacaattagcgcaggaa-3’ |
|  |  | reverse 5’-tgaatgaagccaactgtcgt-3’ |
|  | *daxx* | forward 5’-ttccatgacgtgatgatgtg-3’ |
|  |  | reverse 5’-tctccataagatcccgttcc-3’ |
| Purine metabolism  Pyrimidine metabolism | *pole4* | forward 5’-tgaagaagaatgccacgaag-3’ |
|  |  | reverse 5’-catttctgtcgctttagtaacca-3’ |
|  | *pole2* | forward 5’-cacctgtggctatcgtgttc-3’ |
|  |  | reverse 5’-tgtattctgcatgggttcgt-3’ |
|  | *rpb5* | forward 5’-gggaatgtcaccatcagcta-3’ |
|  |  | reverse 5’-cttgccaagagttcttgcttt-3’ |
|  | *rpc10* | forward 5’-aagaagctgatgtgggtcct-3’ |
|  |  | reverse 5’-ttacctctggcagcaatctg-3’ |
|  | *rpb4* | forward 5’-tatgtggtggaagaggatgc-3’ |
|  |  | reverse 5’-tccggtgtttcaggacataa-3’ |
|  | *rpc37* | forward 5’-caaatgaggccgtcatacac-3’ |
|  |  | reverse 5’-taccaaggctctgcagtcac-3’ |
|  | *apf* | forward 5’-aaagaagaagccggtctgtg-3’ |
|  |  | reverse 5’-cgcaagcaattgtctcatgt-3’ |
|  | *itpa* | forward 5’-taaattgaagccggaaggac-3’ |
|  |  | reverse 5’-tgctggttggaagacacaat-3’ |
|  | *rpc25* | forward 5’-aaccgatcaagcatgggtat-3’ |
|  |  | reverse 5’-tcttgatctggtgtctccca-3’ |
|  | *nt5e* | forward 5’-catacacggaggtgccatag-3’ |
|  |  | reverse 5’-ttgcagaaatcttccagacg-3’ |
| Purine metabolism | *adk* | forward 5’-gcgcacgataatggacatac-3’ |
|  |  | reverse 5’-gtaaagcgatctcctgcaca-3’ |
|  | *allc* | forward 5’-agtcacagtgctggtggaaa-3’ |
|  |  | reverse 5’-cttgatgatgcaccagtcgt-3’ |
|  | *prps* | forward 5’-cacttgctacgacgtctggt-3’ |
|  |  | reverse 5’-tcaacgcacgtttccatatt-3’ |
|  | *pde* | forward 5’-atcactgacgaccagcagag-3’ |
|  |  | reverse 5’-catgattggatctggagcag-3’ |
|  | *gart* | forward 5’-ctgccctcgataaagaaagg-3’ |
|  |  | reverse 5’-accagctcagggtcagctat-3’ |
| Pyrimidine metabolism | *urh1* | forward 5’-agcgcaagccctgatatag-3’t |
|  |  | reverse 5’-gtccagagctatggcaacaa-3’ |
